# Endothelial mineralocorticoid receptor contributes to systolic dysfunction induced by pressure overload without modulating cardiac hypertrophy or inflammation

**DOI:** 10.14814/phy2.13313

**Published:** 2017-06-22

**Authors:** Ane M. Salvador, M. Elizabeth Moss, Mark Aronovitz, Kathleen B. Mueller, Robert M. Blanton, Iris Z. Jaffe, Pilar Alcaide

**Affiliations:** ^1^ Department of Integrative Physiology and PathobiologyTufts University School of MedicineBostonMassachusetts; ^2^ Sackler School of Biomedical SciencesTufts University School of MedicineBostonMassachusetts; ^3^ Molecular Cardiology Research InstituteTufts Medical CenterBostonMassachusetts; ^4^ Centro de Investigaciόn BiomédicaUniversidad de GranadaSpain

**Keywords:** Adhesion molecules, endothelial cell, heart failure, inflammation, mineralocorticoid receptor

## Abstract

Heart Failure (HF) is associated with increased circulating levels of aldosterone and systemic inflammation. Mineralocorticoid receptor (MR) antagonists block aldosterone action and decrease mortality in patients with congestive HF. However, the molecular mechanisms underlying the therapeutic benefits of MR antagonists remain unclear. MR is expressed in all cell types in the heart, including the endothelial cells (EC), in which aldosterone induces the expression of intercellular adhesion molecule 1 (ICAM‐1). Recently, we reported that ICAM‐1 regulates cardiac inflammation and cardiac function in mice subjected to transverse aortic constriction (TAC). Whether MR specifically in endothelial cells (EC) contributes to the several mechanisms of pathological cardiac remodeling and cardiac dysfunction remains unclear. Basal cardiac function and LV dimensions were comparable in mice with MR selectively deleted from ECs (EC‐MR ^−/−^) and wild‐type littermate controls (EC‐MR ^+/+^). MR was specifically deleted in heart EC, and in EC‐containing tissues, but not in leukocytes of TAC EC‐MR ^−/−^ mice. While EC‐MR ^−/−^ TAC mice showed preserved systolic function and some alterations in the expression of fetal genes, the proinflammatory cytokine TNF *α* and the endothelin receptors in the LV as compared to EC‐MR ^+/+^ TAC mice, no difference was observed between both TAC groups in overall cardiac hypertrophy, ICAM‐1 LV expression and leukocyte infiltration, cardiac fibrosis or capillary rarefaction, all hallmarks of pathological cardiac remodeling. Our data indicate that EC‐MR contributes to the transition of cardiac hypertrophy to systolic dysfunction independently of other maladaptive changes induced by LV pressure overload.

## Introduction

Heart failure (HF) is the leading cause of hospitalization in the United States, and an important cause of mortality worldwide (Braunwald 2013), therefore a better understanding of the molecular mechanisms driving HF is needed to mitigate the associated morbidity and mortality. Serum levels of the hormone aldosterone are increased in patients with HF with both reduced and preserved ejection fraction (HFrEF and HFpEF, respectively), and predict mortality risk (Guder et al. 2007; Girerd et al. 2013). Aldosterone functions by binding to the mineralocorticoid receptor (MR) in the kidney to regulate sodium balance (Rogerson and Fuller 2000) and in smooth muscle cells to regulate vasoconstriction (McCurley et al. 2012) thereby contributing to volume overload and increased systemic vascular resistance, important contributors to HF pathophysiology. MR is additionally expressed and functional in multiple cells in the heart, including cardiac myocytes (Lombes et al. 1995), fibroblasts (Bunda et al. 2009), vascular smooth muscle cells (Jaffe and Mendelsohn 2005), endothelial cells (EC) (Caprio et al. 2008), and infiltrated immune cells (Bene et al. 2014). Multiple large randomized trials have demonstrated that MR antagonism with spironolactone or eplerenone, reduces blood pressure in hypertensives and increases survival in patients with HFrEF (Pitt et al. 1999, 2003; Zannad et al. 2011). In patients with HFpEF, a role for MR is more controversial as the recent TOPCAT trial showed no overall benefit to MR inhibition (Pitt et al. 2014). However, secondary and post hoc analyses revealed that MR antagonism improved quality of life in patients with HFpEF (Lewis et al. 2016) and reduced hospitalization and mortality in those enrolled in the Americas (rather than Eastern Europe) (Pfeffer et al. 2015; Bristow et al. 2016) supporting the hypothesis that in subpopulations of patients with HFpEF, MR may play a role in disease progression.

Based on these clinical studies, investigators have been dissecting the role of MR in the progression of HF using tissue‐specific MR knockout (KO) mouse models to determine the role of MR in each cell type in mice subjected to HF induced by hypertension or direct pressure overload by transverse aortic constriction (TAC). In response to pressure overload, the heart undergoes a process of cardiac inflammation, pathologic hypertrophy, and cardiac fibrosis with associated diastolic dysfunction, which contributes to the pathogenesis of HFpEF. Over time, this progresses to cardiac dilation and systolic dysfunction and HFrEF. Studies using the TAC model, which reproduces this progression of cardiac remodeling leading to failure, demonstrated that specific deletion of MR from cardiac myocytes, but not cardiac fibroblasts, preserved systolic function despite no changes in pathological cardiac hypertrophy and fibrosis in response to left ventricular (LV) pressure overload (Lother et al. 2011). Studies using the hypertension model induced with the mouse mineralocorticoid deoxycorticosterone (DOCA) and salt, reported that myeloid cell‐specific MR knockout mice and mice with conditional deletion of MR in both myeloid cells and endothelial cells (EC), were protected from cardiac fibrosis, with the latter model also showing signs of decreased cardiac inflammation (Rickard et al. 2009; Shen et al. 2016). However, whether the protection from cardiac fibrosis and inflammation arose through deletion of MR in myeloid cells, ECs, or in both could not be determined as the Tie2Cre recombinase used in this model expresses in both endothelial and myeloid lineages (Rickard et al. 2014). Altogether, these studies suggest that MRs in distinct cell types in the heart differentially contribute to discrete steps in the pathogenesis of cardiac remodeling and HF in response to pressure overload, however, the specific role of MR in endothelial cells remains to be clarified.

Multiple lines of evidence suggest that EC MR contributes to vascular inflammation, with in vitro data supporting that aldosterone induces intercellular adhesion molecule 1 (ICAM‐1) expression and leukocyte adhesion to coronary endothelial cells (Caprio et al. 2008) and that this process contributes to aldosterone‐induced vascular inflammation and atherosclerosis in vivo (McGraw et al. 2013; Marzolla et al. 2017). We recently showed using the TAC model, that ICAM‐1 is necessary for recruitment of T cells and proinflammatory monocytes to the heart in response to pressure overload and that this mechanism is necessary for progression to systolic dysfunction and HF (Nevers et al. 2015; Salvador et al. 2016). Interestingly, in that study, deletion of EC‐MR did not affect cardiac ICAM‐1 protein expression in response to pressure overload. Moreover, a recent report shows that endothelial cells constitute the majority of noncardiomyocytes in the heart and thus may play a greater role in cardiac physiology and response to injury than previously appreciated (Pinto et al. 2016). However, the role of EC‐MR in the progression of cardiac remodeling and dysfunction in response to pressure overload is not clear. We hypothesized that EC‐MR contributes to adverse cardiac remodeling and HF by enhancing proinflammatory leukocyte recruitment in the LV and cardiac inflammation as it does in the vasculature (McGraw et al. 2013). To test this hypothesis, we subjected mice with MR specifically deleted from ECs (EC‐MR^−/−)^, but intact in leukocytes, to TAC and compared the cardiac response to their EC‐MR‐intact littermates (EC‐MR^+/+^). We previously reported that these EC‐MR^−/−^ male mice are not different from EC‐MR^+/+^ littermates in systolic, diastolic, circadian, and salt‐sensitive blood pressure, as determined by telemetric blood studies (Mueller et al. 2015). Additionally, coronary arterioles from EC‐MR^−/−^ mice have normal vasorelaxation capacity at baseline with enhanced vasoconstriction to endothelin and thromboxane, as determined by vessel myography studies (Mueller et al. 2015). Here we report that deletion of EC‐MR does not affect cardiac function in unchallenged mice. In response to TAC, deletion of EC‐MR preserves systolic function, without affecting cardiac hypertrophy, fibrosis, and capillary rarefaction. Contrary to our hypothesis, EC‐MR deficiency does not alter cardiac inflammatory cell infiltration in response to TAC but rather LV expression of cardiac TNF*α*, natriuretic peptides, and endothelin receptors is significantly reduced in EC‐MR^−/−^ mice as compared to EC‐MR^+/+^ littermate control mice after exposure to TAC. Thus, our data indicate that EC‐MR does not contribute to hypertrophy in response to pressure overload but rather promotes the transition to systolic dysfunction independently of LV recruitment of immune cells by a mechanism that may involve regulation of coronary vascular function through regulation of vasoconstrictive pathways.

## Methods

### Mice

Mice were bred and maintained under pathogen‐free conditions and treated in compliance with the *Guide for the Care and Use of Laboratory Animals* (National Academy of Science). All experiments were conducted using male mice on the C57Bl/6 background. Mice with loxP sites flanking exons 5 and 6 of the MR gene (MRf/f) (McCurley et al. 2012) were bred with mice containing a Cre recombinase transgene driven by the EC‐specific VE‐cadherin promoter (Cre+) (Alva et al. 2006). Endothelial cell specific mineralocorticoid receptor (MR) knock‐out (EC‐MR^−/−^) and MR intact (EC‐MR^+/+^) male littermates underwent TAC surgery at 8–10 weeks of age and were euthanized at 12–14 weeks of age for tissue collection; All protocols were approved by the Tufts University and Tufts Medical Center Institutional Animal Care and Use Committee.

### Isolation of primary mouse heart endothelial cells (MHEC)

Mouse coronary ECs were isolated from hearts harvested from five to eight mice at 2–4 weeks of age as previously described (Mueller et al. 2015). Briefly, hearts were digested in 2 mg/mL of collagenase shaking at 37°C for 30 min, and incubated with PECAM‐1‐coated sheep anti‐rat‐IgG Dynabeads (15 *μ*L/mL of cells) at room temperature for 10 min with end‐over‐end rotation. Magnetically recovered cells were resuspended in 10 mL of complete culture medium, (DMEM‐20% supplemented with 100 *μ*g/mL heparin, 100 *μ*g/mL ECGS, NEAA, sodium pyruvate, L‐glutamine, and Pen‐Strep, at standard concentrations), and plated in two gelatin‐coated 65‐mm dishes. When 75–85% cell confluence was reached, they were sorted again with ICAM‐2‐coated sheep anti‐rat‐IgG Dynabeads beads (15 *μ*L/mL of cells). Bead‐bound cells were washed and plated in complete DMEM culture medium and passaged further at a 1:3 ratio. Mouse heart endothelial cell isolates were used at passages 2–4 for experiments, and purified as previously described (Alcaide et al. 2012; Mueller et al. 2015).

### PCR analysis of Nr3c2 genomic DNA to confirm EC‐specific recombination after TAC

Right ventricle, lungs, and lymph nodes were isolated from EC‐MR^−/−^ and MR‐intact littermates at the time of sacrifice and frozen in liquid nitrogen. DNA was extracted from each tissue with the DNeasy kit (Qiagen) and PCR was performed as previously described (McCurley et al. 2012) using a combination of three primers: MR‐4 CCA CTT GTA TCG GCA ATA CAG TTT AGT GTC; MR‐5 CAC ATT GCA TGG GGA CAA CTG ACT TC; MR‐3 CTG TGA TGC GCT CGG AAA CGG. PCR products were run on a 1.5% agarose gel.

### Mouse model of transverse aortic constriction

Pressure overload was induced by constricting the transverse aorta to the diameter of a 25 gauge needle in 8–10 weeks old EC‐MR^+/+^ and EC‐MR^−/−^ male mice as previously described (Patten et al. 2008; Chintalgattu et al. 2010). Sham‐operated EC‐MR^+/+^ and EC‐MR^−/−^ mice underwent the same procedure but without constriction. 4 weeks after TAC, mice were euthanized and tissues were harvested for further analysis as described (Nevers et al. 2015; Salvador et al. 2016). Processing and analysis of the Sham and TAC groups were done in four different harvest days: 2 days harvesting 1 Sham/2 TAC of each genotype, 1 day harvesting 1 Sham/2 TAC EC‐MR^+/+^ and 2 TAC EC‐MR^−/−^, and a fourth day harvesting 2 Sham and 2 TAC EC‐MR^+/+^, and 1 Sham and 2 TAC EC‐MR^−/−^. None of the mice undergoing surgery died during TAC before the tissue harvest time 4 weeks post TAC.

### LV in vivo hemodynamic studies

In vivo LV function was assessed by pressure–volume analysis in mice anesthetized with 2.5% isofluorane. A 1.4‐French PV catheter (SPR‐839; Millar Instruments) was advanced through the carotid artery and across the aortic valve into the LV. The absolute volume was calibrated, and PV data were assessed at steady state and during preload reduction. Hemodynamics were recorded and analyzed with IOX version 1.8.11 software (EMKA Instruments, Falls Church, VA) (Blanton et al. 2012). Investigators were blinded to genotype and treatment group during performance of hemodynamic studies and analysis of data.

### In vivo transverse echocardiography

M Mode and two‐dimensional images were obtained from the short‐axis view, as described previously (Patten et al. 2008; Blanton et al. 2012). Images were obtained using the VisualSonics 2100 and M Mode images were analyzed using VisualSonics analysis software. Left ventricular end diastolic and end systolic diameters (EDDs and ESDs, respectively) and heart rate were measured by averaging values obtained from 10 cardiac cycles. Fractional shortening was calculated using the following standard equation: FS% = ([EDD−ESD]/EDD)×100. For calculation of LV ejection fraction, ventricular volumes were calculated from M mode dimensions using the equations End Diastolic Volume = (7.0/[2.4 + EDD])*(EDD)^3^ and End Systolic Volume = (7.0/[2.4 + ESD])*(ESD)^3^. Ejection fraction (EF) was then determined using the equation EF = ([EDV−ESV]/EDV)×100.

### Real‐time quantitative polymerase chain reaction (RT‐PCR) of MHEC and LV samples

Total RNA was extracted from MHEC and mouse heart LV tissues directly using Trizol (Invitrogen). RNA was then reverse‐transcribed to cDNA following Applied Biosystems' protocol using MuLV Reverse Transcriptase, and amplified by real‐time PCR with SYBR^®^ green PCR mix (Applied Biosystems). Samples were quantified in triplicate using 40 cycles performed at 94°C for 30 sec, 60°C for 45 sec, 72°C for 45 sec using an ABI Prism^®^ 7900 Sequence Detection System. The sequences of the primers used are the following: forward 5′ – GAA GAG CCC CTC TGT TTG CAG ‐3′, reverse 5′ – TCC TTG AGT GAT GGG ACT GTG ‐3′; *Nr3C2*: forward 5′ – GCT ATC CAG AAA ACC CCT CAA ‐3′, reverse 5′ – CAT GTC TCG ATC CCA GTA GAC GGT ‐3′; *B2MG*: forward 5′ ‐CAC CGC TGG GAG GTC ACT ‐3′, reverse 5′ ‐GTG AGG CCT TGG TCC TTC AA ‐3′; *nppb*: forward 5′‐CCT AGC CAA CTC CCC GTT CT ‐3′, reverse 5′ –GCC AAT GAG TAC CGC GTG A ‐3′; *myh6:* forward 5′‐TGA GCC TTG GAT TCT CAA ACG T ‐3′, reverse 5′ –AGG TGG CTC CGA GAA AGG AA ‐3′; *myh7*: forward 5′ ‐GCT GTG CTT TGA GAA CTG TG ‐3′, reverse 5′ ‐GTG AGG TCC TTG CCT ACT TG ‐3′; *icam1*: forward 5′ –CCA AAT CCA CGC TTG TGT TGA ‐3′, reverse 5′ –GGA ATG AGT AGA CCT CCA CCT ‐3′; *vcam1*: forward 5′‐ ACT CCT TAG TCC TCG GCC A –3′, reverse 5′‐TGG TTT CTT GTG ACC CTG AGC ‐3′; *il1b*: forward 5′ –TGA TGG ATG CTA CCA AAC TGG‐3′, reverse 5′‐TTC ATG TAC TCC AGG TAG CTA TGG ‐3′; *il6*: forward 5′ –GCA CAG AAA GCA TGA CCC G ‐3′, reverse 5′ –GCC CCC CAT CTT TTG GG ‐3′; *tnfa*: forward 5′ –GTG GCT TCT TGG GGG TAT GG ‐3′, reverse 5′ –TCT TAG TGG GTG GCG TCA TTA ‐3′; *ednrb*: forward 5′ –CTG AAA ACA ATT TTT GAA TTT CTT GC‐3′, reverse 5′ –TAC CAA GAT GTG AAG GAC TGG TGG‐3′; *ednra*: forward 5′ –GTG TCT ACT TCT GCC ACC TGG ACA T‐3′, reverse 5′‐ GGG CTC GCA CTA TAT AAG GGA TGA C ‐3′; *edn1*: forward 5′‐ACC ACA GTC CAT GCC ATC AC ‐3′, reverse 5′ –TCC ACC ACC CTG TTG CTG TA – 3′; *gapdh*.

### Immunohistochemistry and histological analysis

Heart samples were excised and the LV was separated from the right ventricle. Sections of the LV were immediately embedded in OCT or fixed in 10% formalin, embedded in paraffin, and cut into 5 *μ*m LV sections. Hematoxylin and Eosin or Picrosirius red staining was performed as described (Herrada et al. 2010; Gueret et al. 2016). Cardiomyocyte size was determined in H&E stained LV cross sections by measuring the area of 10–20 myocytes per mouse LV using the NIS‐Elements AR 4.51.00 software. Collagen deposition was quantified in Picrosirius red stained LV cross sections by determining the intensity of the staining in 5–7 representative fields per LV using Image J. Percent leukocyte infiltration was quantified using Image J by dividing each 40× representative picture in 30 grids and counting the number of grids containing leukocytes infiltrated (% = positive grids/30 × 100). Serial OCT frozen cryostat sections were stained with anti‐mouse CD31 (MEC13.3), anti‐mouse CD4 (GK1.5), anti‐mouse CD11b (M1/70) or anti‐mouse Gr1 (RB6‐8C5) purchased from BioLegend (San Diego, CA). Leukocyte infiltrates were quantified by a blinded investigator counting number of cells positive for each staining per section. Apoptotic cells in frozen sections were stained using ApopTag^®^ Fluoriscein In Situ Apoptosis Detection Kit S7110 (Millipore) following manufacturer's indications.

### Statistical analysis

Data are expressed as the mean ± SEM. Data acquisition and analysis were done by genotype and treatment blinded investigators. Statistical analyses were done by checking the variance among groups using *F*‐statistic and subsequently reanalyzing data that exhibited significant differences in variance using unequal variance *t*‐test for data distributed normally. Data normality was assessed through D'Agostino & Pearson normality test. Data that did not satisfy the assumption of normality, were logarithmically transformed and the equal or unequal variance *t*‐test was used (based on the *F*‐statistic). Unpaired Student *t*‐test or nonparametric Mann–Whitney test were performed, where indicated, using GraphPad Prism software (GraphPad). Differences were considered statistically significant at *P* ≤ 0.05 and are indicated with an (*). ns *P* > 0.05; *P ≤ 0.05; **P ≤ 0.01; ***P ≤ 0.001.

## Results

### A mouse model with MR selectively deleted from ECs but not leukocytes has normal baseline cardiac function

**Figure 1:**
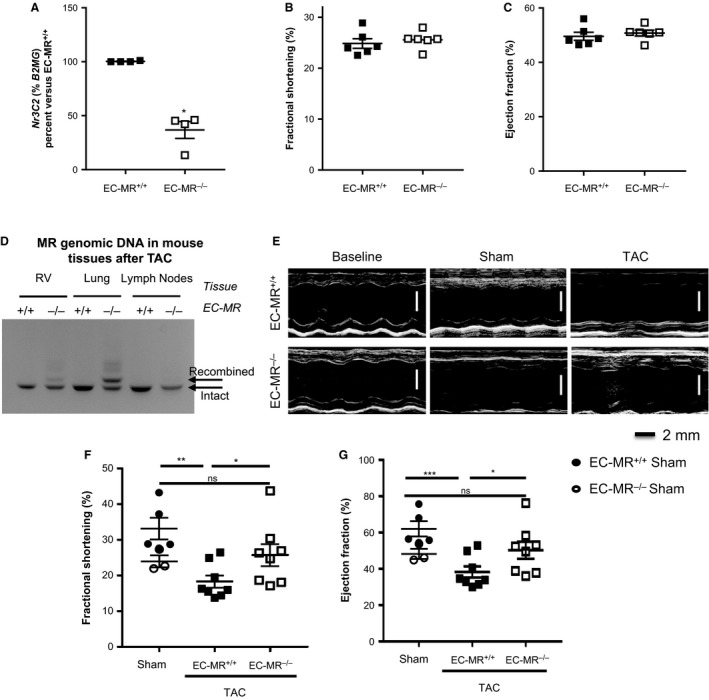
EC‐MR contributes to LV systolic dysfunction in response to TAC. (A) MR recombination in MHEC isolated from EC‐MR ^+/+^ and EC‐MR ^−/−^ mice determined by measuring mRNA expression by qRT‐PCR. Ct values were normalized to *β*2‐microglobulin (B2MG) and mRNA levels in EC‐MR ^−/−^ MHEC were expressed as a percentage of those in EC‐MR ^+/+^ MHEC. (B and C) Baseline echocardiography measurements of the (B) % Fractional Shortening (%FS) and (C) % Ejection Fraction (%EF) of EC‐MR ^+/+^ and EC‐MR ^−/−^ mice. *n* = 6 EC‐MR ^+/+^ and 6 EC‐MR ^−/−^. (D) MR recombination in VE‐Cad‐Cre+ EC‐MR ^−/−^ mice specifically in endothelial cell (EC)‐containing tissues (heart and lung) but not in leukocytes (lymph node cells) 4 weeks after TAC. RV, right ventricle; MR, mineralocorticoid receptor. (E) Representative echocardiography images of baseline, Sham and 4 week operated TAC EC‐MR ^+/+^ and EC‐MR ^−/−^ mice. (F) %FS and (G) %EF measured by noninvasive echocardiography. *n* = 5 EC‐MR ^+/+^ Sham, 8 EC‐MR ^+/+^ TAC, 3 EC‐MR ^−/−^ Sham, 8 EC‐MR ^−/−^ TAC. %FS is calculated by [(LVEDD‐LVESD)/LVEDD]*100. Statistics D'Agostino & Pearson normality test, followed by unpaired Student *t*‐test.

We previously generated a mouse with the VE‐Cadherin promoter driving Cre recombinase crossed to the floxed MR mouse and demonstrated specific recombination of the MR gene in EC‐containing tissues including heart, lung, and vessels, and in isolated endothelial cells, with no recombination in immune cells (Mueller et al. 2015). Cardiac EC‐MR deficiency was confirmed by isolating mouse heart endothelial cells (MHECs) from both EC‐MR^+/+^ and EC‐MR^−/−^ mice and the abundance of MR (Nr3C2 gene) mRNA was quantified by PCR. We observed that mRNA derived from VE‐Cad‐Cre+ cultured ECs had a 70% decrease in MR expression compared to Cre‐ MHEC cultures (Fig. 1A). The remaining MR expression is interpreted as a combination of incomplete recombination or possibly slight contamination of the culture with non‐ECs such as fibroblasts and smooth muscle cells (Alcaide et al. 2012) that also express MR. We previously reported that these EC‐MR^−/−^ mice display no basal defect in growth, vascular relaxation function, or blood pressure (Mueller et al. 2015). We next performed transverse echocardiography on 10–12‐week‐old adult male EC‐MR^−/−^ and EC‐MR^+/+^ littermates to assess baseline cardiac function. There were no differences in basal LV structure or systolic and diastolic function between genotypes (Fig. 1B–C and Table 1). We next subjected male EC‐MR^−/−^ and EC‐MR^+/+^ intact littermates to TAC in order to explore the specific role of EC‐MR in the cardiac remodeling response to pressure overload. We confirmed selective recombination of the MR gene from EC‐containing tissues (right ventricle‐RV and lung tissue) 4 weeks after TAC only in Cre positive EC‐MR^−/−^ mice by PCR (Fig. 1D, upper band). Importantly, after TAC, there was no MR recombination in DNA from leukocytes within inguinal and axillary lymph nodes of mice subjected to TAC. The intensity of the recombined band in EC‐MR^−/−^ was proportional to the expected presence of ECs in each organ with more recombination in the lungs than the heart and no recombination in DNA isolated from the lymph nodes of the same mice (Fig. 1D). Thus, this model enables selective exploration of the specific role of EC‐MR in cardiac remodeling and function in response to pressure overload in the presence of intact leukocyte MR.

**Table 1:** Baseline cardiac function characterization through echocardiographic studies

	EC‐MR^+/+^	EC‐MR^−/−^
Anterior wall thickness (mm)	0.8647 ± 0.044	0.841 ± 0.045
Posterior wall thickness (mm)	0.7638 ± 0.022	0.8165 ± 0.065
End diastolic diameter (mm)	4.137 ± 0.163	4.195 ± 0.049
End systolic diameter (mm)	3.106 ± 0.118	3.087 ± 0.039
Fractional shortening (%)	24.86 ± 0.944	25.57 ± 0.686
Ejection fraction (%)	49.6 ± 1.469	50.77 ± 1.087
Heart rate (bpm)	433.5 ± 19.07	423.3 ± 8.713

*n* = 6 EC‐MR^+/+^ and 6 EC‐MR^−/−^. Mean ± SEM is indicated for each parameter.

### Mice lacking MR in ECs are protected from the decline in systolic function in response to TAC

Four weeks after Sham or TAC surgery, LV function was assessed by echocardiography and invasive hemodynamic analysis. TAC induced equivalent increases in LV pressure overload in both EC‐MR^+/+^ and EC‐MR^−/−^ littermates as compared to their Sham‐operated EC‐MR^+/+^ and EC‐MR^−/−^ controls. Specifically, LV systolic pressure and arterial elastance (Ea) were not different in the TAC groups (Table 2). Complete LV hemodynamic data are presented in Table 2. As expected, echocardiography revealed that in EC‐MR^+/+^ mice, LV fractional shortening, and ejection fraction, indices of systolic function, began to decline 4 weeks after TAC. In contrast, EC‐MR^−/−^ mice had preserved ejection fraction and percent fractional shortening compared to EC‐MR^+/+^ exposed to same degree and duration of TAC, and similar to Sham EC‐MR^−/−^ controls (Fig. 1E–G). We also noticed that end systolic diameter (ESD) trended to a decrease in Sham‐operated mice (Table 2) as compared to mice which had no surgery (Table 1), however, it was significantly increased, as expected, in response to TAC in EC‐MR^+/+^ mice in contrast to EC‐MR^−/−^ TAC mice which did not show such increase (Table 2). However, indices of diastolic function, including d*P*/d*t*_min_ and tau (time constant of LV relaxation), did not differ between genotypes. The decline in systolic function in EC‐MR^+/+^ mice was associated with a significant increase in LV end diastolic pressure in EC‐MR^+/+^ TAC mice compared with EC‐MR^+/+^ Sham controls (Table 2), indicating LV decompensation, an early sign of the development of the clinical syndrome of heart failure. While LV end diastolic pressure also rose in the EC‐MR^−/−^ TAC mice, this was not significantly different from Sham EC‐MR^−/−^ mice suggesting that the preservation of systolic function also attenuated the early progression to HF. As expected at this early stage of HF progression only 4 weeks after TAC, pulmonary edema had not yet developed, as evidenced by no change in lung weight (Table 2). Thus, these findings demonstrate that deletion of EC‐MR improves indices of systolic function but not diastolic function in response to pressure overload.

**Table 2:** Cardiac function characterization through hemodynamic and echocardiographic studies, and lung weight

	EC‐MR^+/+^ TAC		EC‐MR^−/−^ TAC	
Lung weight	Sham 8.59 ± 0.19	TAC 8.65 ± 0.2	Sham 8.58 ± 0.06	TAC 8.92 ± 0.19
Hemodynamics				
Systolic pressure (mmHg)	106.2 ± 2.676	164.5 ± 4.435***	105.4 ± 3.925	163 ± 8.4**
End diastolic pressure (mmHg)	4.44 ± 0.74	14.86 ± 2.834^a^	5.267 ± 1.317	13.13 ± 3.506
Effective arterial elastance (mmHg/*μ*L)	8.776 ± 2.03	11.27 ± 1.041	8.22 ± 2.934	11.98 ± 1.482
dP/dt_max_	9119 ± 468.3	8235 ± 653.1	8861 ± 728.2	7818 ± 761.7
dP/dt_min_	−9715 ± 793.2	−8985 ± 1043	−8940 ± 670.7	−8863 ± 655
Tau " (msec)	4.54 ± 0.21	4.625 ± 0.30	4.367 ± 0.37	4.65 ± 0.237
End diastolic volume (*μ*L)	35.78 ± 9.259	42.13 ± 5.101	35.8 ± 11.1	36.79 ± 4.56
End systolic volume (*μ*L)	25.11 ± 5.786	31.91 ± 3.811	23.99 ± 5.615	30.1 ± 3.881
Cardiac output (*μ*L/min)	7544 ± 1890	7136 ± 558.5	7475 ± 2085	5810 ± 815.7
ESPVR (mmHg/*μ*L)	4.078 ± 0.892	4.888 ± 0.763	3.65 ± 0.496	6.135 ± 1.355
PRSW (mmHg/*μ*L)	44.9 ± 11.73	70.02 ± 10.17	65.14 ± 9.932	81.09 ± 16.05
Heart rate (bpm)	490.4 ± 17.57	493.9 ± 10.34	481.7 ± 45.08	449.3 ± 21.27
Echocardiography				
End diastolic diameter (mm)	3.88 ± 0.14	3.92 ± 0.12	3.946 ± 0.10	4.05 ± 0.14
End systolic diameter (mm)	2.61 ± 0.19	3.21 ± 0.12	2.99 ± 0.01	3.03 ± 0.21

*n* = 5 EC‐MR^+/+^ Sham, 8 EC‐MR^+/+^ TAC, 3 EC‐MR^−/−^ Sham, 8 EC‐MR^−/−^ TAC. Mean ± SEM is indicated for each parameter. Statistics D'Agostino & Pearson normality test, followed by unpaired Student's *t*‐test. **P < 0.01; ***P < 0.005.

ESPVR, end systolic pressure–volume relationship; PRSW, preload recruitable stroke work.

a Versus Sham per genotype.

### EC‐MR does not contribute to LV hypertrophy in response to TAC

**Figure 2:**
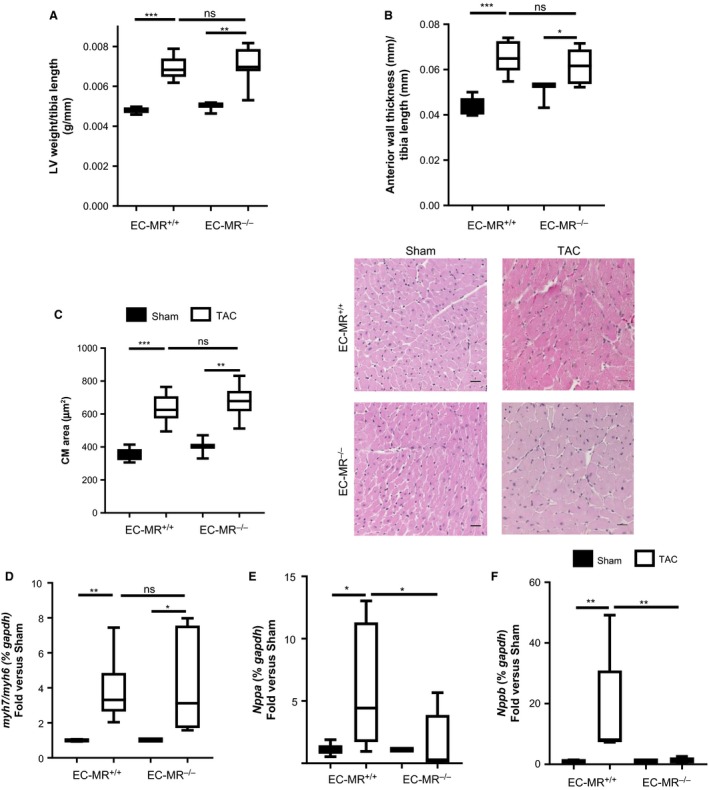
EC‐MR does not contribute to LV or cardiac myocyte hypertrophy but play a role in the release of natriuretic peptides in response to TAC. (A) LV weight normalized with the tibia length (g/mm). (B) LV relative anterior wall thicknesses determined by noninvasive echocardiography normalized to tibia length. (C) Cardiac Myocyte (CM) area measured in H&E stained LV cross sections; quantification is shown on the left and 40 ×  representative pictures are shown on the right. Statistics D'Agostino & Pearson normality test, followed by unpaired Student's *t*‐test. LV (D) Ratio of mRNA levels of MHC *β* isoform over MHC *α*, represented as fold versus respective Sham. (E) ANP and (F) BNP mRNA levels, represented as fold versus respective Sham. *n* = 5 EC‐MR ^+/+^ Sham, 8 EC‐MR ^+/+^ TAC, 3 EC‐MR ^−/−^ Sham, 8 EC‐MR ^−/−^ TAC. Statistics, unpaired Mann–Whitney *t*‐test.

We next evaluated cardiac hypertrophy after 4 weeks of TAC as pathological hypertrophy is an early response to pressure overload that contributes to diastolic dysfunction. As expected, LV mass increased in EC‐MR^+/+^ mice in response to TAC compared to Sham surgery. Moreover, LV mass increased to the same extent in EC‐MR^−/−^ and EC‐MR^+/+^ mice in response to TAC (Fig. 2A) consistent with the lack of difference in diastolic function parameters (Table 2). LV anterior wall thickness was significantly increased in both genotypes after TAC as compared to Sham controls, further confirming the similar degree of hypertrophy as no difference was observed between genotypes after TAC (Fig. 2B). Moreover, LV cross sections stained with H&E showed an increase in CM area in TAC mice compared to Sham, that did not differ between EC‐MR^−/−^ and EC‐MR^+/+^ littermates (Fig. 2C). Overall, these data indicate that EC‐MR does not appear to play a role in LV or CM hypertrophy in response to pressure overload induced by TAC. We also compared fetal gene expression, which represents a molecular signature correlating with pathologic, as opposed to adaptive, cardiac hypertrophy. In response to TAC, both EC‐MR^+/+^ and EC‐MR^−/−^ LV showed an isoform switch from adult to fetal myosin heavy chain (MHC*α* to MHC*β*) that resulted in an increased MHC*β*/*α* ratio in TAC mice relative to Sham controls, in which the switch to MHC*β* did not occur (Fig. 2D). However, while EC‐MR^+/+^ exhibited an expected increase in mRNA level of the natriuretic peptides ANP (Fig. 2E) and BNP (Fig. 2F) in TAC versus Sham, there was no such increase observed in these genes in EC‐MR^−/−^ after TAC, and the expression of ANP and BNP were significantly decreased in EC‐MR^−/−^ compared to EC‐MR^+/+^ mice after TAC. Taken together, these data confirm the induction of pathologic LV hypertrophy in this model and further reveal that EC‐MR^−/−^ mice are not protected from pressure overload‐induced LV hypertrophy. However, since ANP and BNP expression are upregulated in response to atrial stretch, the differences between EC‐MR^−/−^ and EC‐MR^+/+^ may reflect the protection from LV systolic dysfunction and associated increases in LV end diastolic pressure.

### EC‐MR is not essential for the expression of cardiac adhesion molecules and leukocyte recruitment in response to TAC

**Figure 3:**
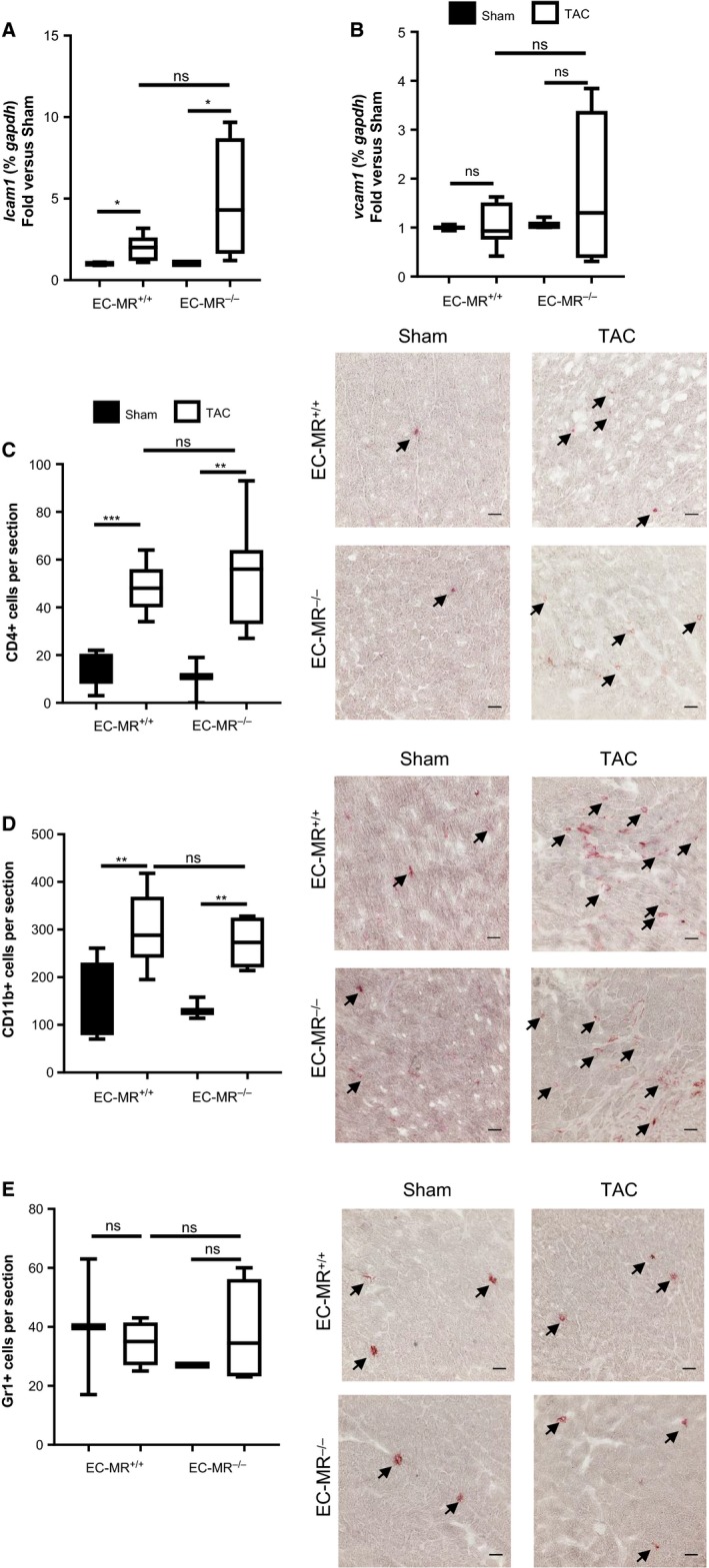
EC‐MR does not regulate endothelial cell adhesion molecule expression, CD4 +  T cell and CD11b myeloid cell recruitment in response to TAC. (A) ICAM‐1 and (B) VCAM‐1 mRNA levels represented as fold versus respective Sham. Statistics, unpaired Mann–Whitney *t*‐test. (C) LV CD4 +  T lymphocyte recruitment, (D) CD11b+ myeloid cell and (E) Gr1 +  neutrophil recruitment determined by IHC as number of positive cells per section. Arrows indicate representative staining. Quantification of infiltrated cells is shown on the left and representative 40 ×  pictures are shown on the right. Scale bar 25 *μ*m. *n* = 5 EC‐MR ^+/+^ Sham, 8 EC‐MR ^+/+^ TAC, 3 EC‐MR ^−/−^ Sham, 8 EC‐MR ^−/−^ TAC. Statistics D'Agostino & Pearson normality test, followed by unpaired Student's *t*‐test.

Aldosterone and MR have been implicated in vascular and systemic inflammation in hypertension (Caprio et al. 2008; McGraw et al. 2013) and atherosclerosis (McGraw et al. 2013). We have previously reported that MR activation by aldosterone upregulates ICAM‐1 in human coronary ECs (Caprio et al. 2008), and that ICAM‐1 mediates T cell and CD11b+ recruitment into the LV after TAC (Salvador et al. 2016). Thus, we initially hypothesized that EC‐MR would be required for cardiac adhesion molecule expression and proinflammatory leukocyte infiltration as a mechanism responsible for the observed preserved systolic function in EC‐MR^−/−^ mice in response to TAC. As expected, ICAM‐1 mRNA levels increased in EC‐MR^+/+^ LVs in response to TAC. However, ICAM‐1 mRNA levels also increased to the same extent in LV from EC‐MR^−/−^ TAC mice compared to Sham, indicating that ICAM‐1 induction by pressure overload was not affected by deletion of EC‐MR (Fig. 3A). Vascular cell adhesion molecule 1 (VCAM‐1) mRNA expression was not significantly altered in either genotype in response to TAC (Fig. 3B). Immunohistochemistry was performed to visualize infiltrating leukocytes in LV cross sections subjected to 4‐week TAC. Quantification of the staining demonstrated that CD4+ T cells (Fig. 3C) and CD11b+ myeloid cell (Fig. 3D) infiltration was increased in EC‐MR^+/+^ mice in response to TAC and this was unaffected by EC‐MR deletion. Gr1+ neutrophil infiltration did not change between Sham and TAC mice of either genotype (Fig. 3E), as previously demonstrated in WT mice (Nevers et al. 2015). Taken together, these data indicate that the contribution of EC‐MR to systolic dysfunction in response to TAC is independent of cardiac adhesion molecule expression and LV CD4+ T cell and CD11b+ monocyte infiltration.

### EC‐MR does not contribute to cardiac fibrosis in response to TAC

**Figure 4:**
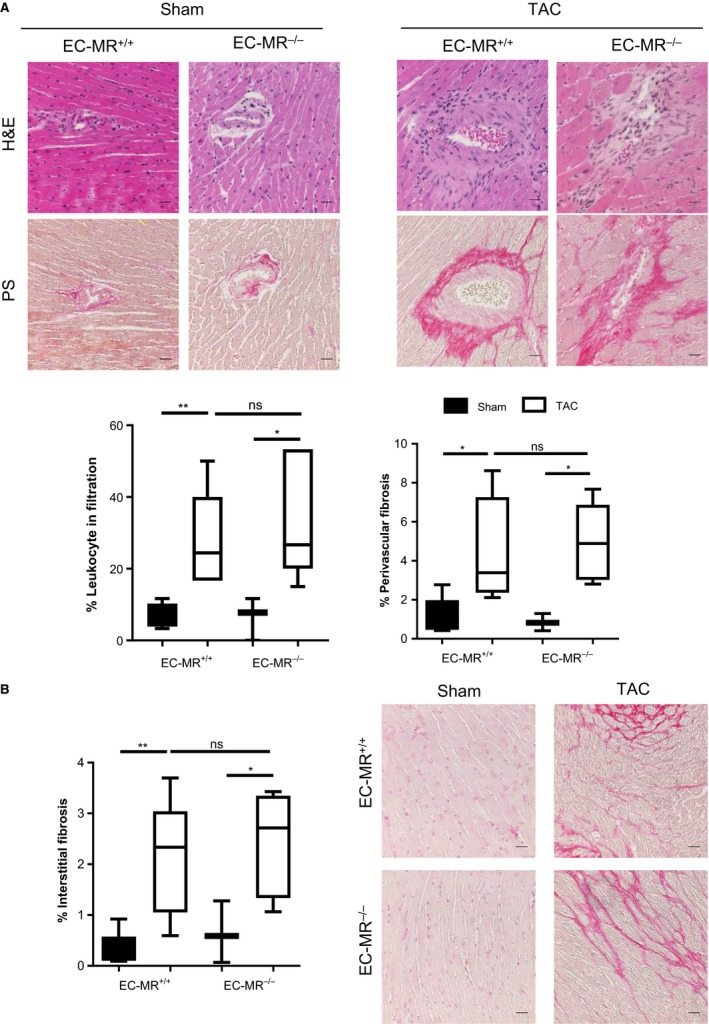
EC‐MR does not regulate perivascular inflammation, perivascular, and interstitial collagen deposition in response to TAC. (A) Representative H&E and Picrosirius red‐stained LV sections, showing areas with elevated leukocyte infiltration colocalizing with areas presenting high percentage of perivascular fibrosis; quantifications of infiltrates and perivascular collagen deposition are shown below. (B) LV interstitial collagen deposition quantified as percent fibrosis on the left. Scale bar 25 *μ*m. *n* = 5 EC‐MR ^+/+^ Sham, 8 EC‐MR ^+/+^ TAC, 3 EC‐MR ^−/−^ Sham, 8 EC‐MR ^−/−^ TAC. Statistics D'Agostino & Pearson normality test, followed by unpaired Student's *t*‐test

Another hallmark of pathological cardiac remodeling in response to TAC is cardiac fibrosis. Moreover, aldosterone promotes perivascular inflammation and fibrosis (Rocha et al. 2002). Thus, we next examined whether EC‐MR regulates cardiac fibrosis as a potential mechanism underlying the preservation of systolic function observed in EC‐MR^−/−^ mice in response to TAC. Fibrosis was determined by quantification of collagen deposition in the LV stained with Picrosirius red. Perivascular fibrosis was first examined and the degree of fibrosis in response to TAC did not differ between EC‐MR^+/+^ and EC‐MR^−/−^mice (Fig. 4A). Interestingly, perivascular areas with high collagen deposition colocalized with significant immune cell infiltration after TAC determined by H&E. However, deletion of EC‐MR also did not affect the degree of perivascular inflammatory leukocyte infiltration (Fig. 4A). Interstitial fibrosis was also similar in EC‐MR^+/+^ mice and EC‐MR^−/−^ in response to TAC (Fig. 4B). Taken together these data support that EC‐MR does not play a significant role in LV interstitial of perivascular fibrosis induced by TAC.

### EC‐MR is dispensable for capillary rarefaction and cardiac apoptosis in response to TAC

**Figure 5:**
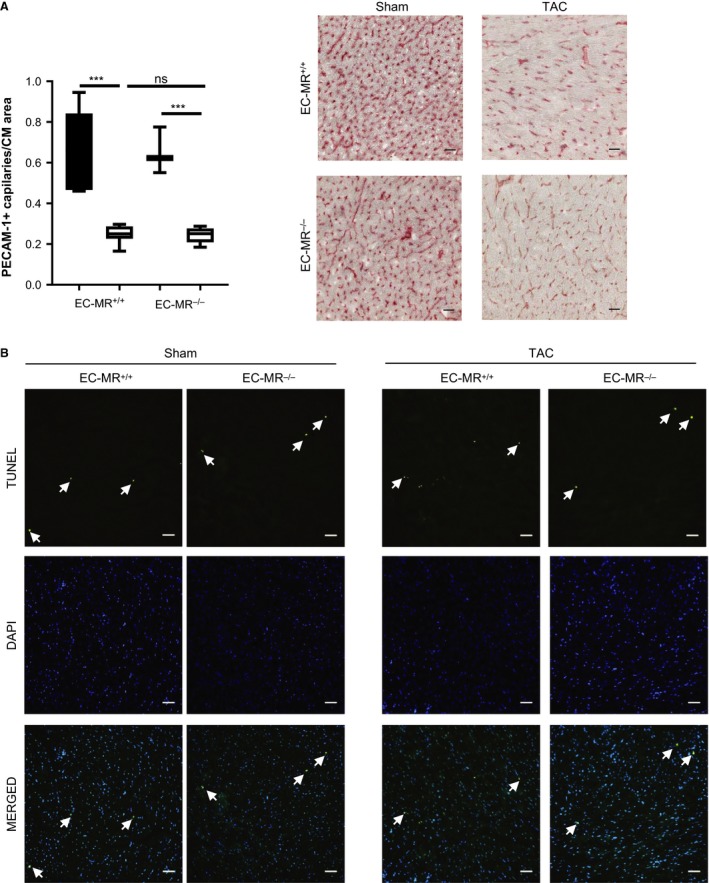
EC‐MR deletion does not affect the degree of angiogenesis and cardiomyocyte apoptosis in response to TAC. (A) LV capillary density, determined by IHC as PECAM1 +  (CD31) capillary number normalized by CM area. Quantification is shown on the left and representative pictures on the right. Scale bar 25 *μ*m. *n* = 5 EC‐MR ^+/+^ Sham, 8 EC‐MR ^+/+^ TAC, 3 EC‐MR ^−/−^ Sham, 8 EC‐MR ^−/−^ TAC. Statistics D'Agostino & Pearson normality test, followed by unpaired Student t‐test. (B) LV CM apoptosis determined using the TUNEL staining. Scale bar 50 *μ*m.

In our search for mechanisms by which EC‐MR contributes to systolic dysfunction, we next sought to determine the role of EC‐MR in cardiac angiogenesis in response to TAC. A decrease in capillary number relative to cardiomyocyte area in the heart represents an additional feature of maladaptive cardiac remodeling that is thought to contribute to the development of LV dysfunction (Tsagalou et al. 2008). We stained LV cross sections with platelet endothelial cell adhesion molecule 1 (PECAM‐1) to quantify intramyocardial capillaries relative to the cardiac myocyte area as a measurement of capillary density in the LV. EC‐MR^−/−^ and EC‐MR^+/+^ both had the same decrease in LV capillary density in response to TAC as compared to Sham control mice (Fig. 5A). We also evaluated the possibility that EC‐MR might contribute to cardiomyocyte apoptosis in response to TAC, as an additional potential mechanism to explain the improved systolic function in EC‐MR^−/−^ mice. TUNEL staining in LV sections indicated that very little apoptosis is occurring in EC‐MR^+/+^ at 4 weeks after TAC, as previously described for WT C57/BL6 mice (Li et al. 2009; Nevers et al. 2015). Moreover, the amount of TUNEL+ cells was comparable between TAC EC‐MR^+/+^ mice and TAC EC‐MR^−/−^ at this time (Fig. 5B). Thus, these data support that EC‐MR does not contribute to systolic dysfunction by playing a significant role in LV capillary rarefaction or cardiomyocyte apoptosis induced by TAC.

### EC‐MR contributes to LV expression of TNF*α* and endothelin receptors in response to TAC

**Figure 6:**
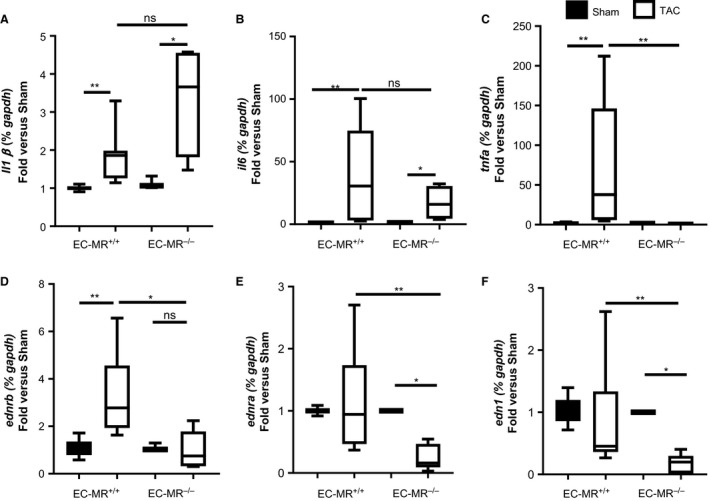
EC‐MR contributes to the LV expression of TNF *α* and endothelin A and B receptors in response to TAC. (A) IL‐1*β*, (B) IL‐6, (C) TNF *α*, (D) endothelin receptor type B, (E) endothelin receptor type A and (F) endothelin‐1 mRNA levels represented as fold versus respective Sham. *n* = 5 EC‐MR ^+/+^ Sham, 8 EC‐MR ^+/+^ TAC, 3 EC‐MR ^−/−^ Sham, 8 EC‐MR ^−/−^ TAC. Statistics, unpaired Mann–Whitney *t*‐test.

We had previously postulated that T‐cell infiltration in the LV in response to TAC drives proinflammatory cytokine production in the LV that contributes to cardiomyocyte dysfunction leading to the development of HF (Nevers et al. 2015). Although TAC EC‐MR^−/−^ mice had similar T‐cell infiltration as their TAC WT littermates, we next evaluated LV expression of proinflammatory cytokines in EC‐MR^−/−^ mice. While IL‐1*β* and IL‐6 were similarly upregulated in the LV in response to TAC compared to Sham control animals in both genotypes (Fig. 6A and B), TNF*α* mRNA expression did not follow the same pattern but rather was increased in TAC EC‐MR^+/+^ but not in TAC EC‐MR^−/−^ mice (Fig. 6C). These data indicate that EC‐MR is essential for upregulation of LV TNF*α* expression in response to TAC. It further suggests the possibility that the lack of myocardial TNF*α* expression may contribute to the ameliorated systolic dysfunction in EC‐MR^−/−^ mice, and supports that IL‐1*β* and IL‐6, but not TNF*α*, are more likely to be the inducers of ICAM‐1, as we have previously reported (Salvador et al. 2016).

Finally, we investigated whether EC‐MR regulates endothelin receptor expression in the heart as a potential mechanism contributing to systolic dysfunction after TAC. Endothelin‐1 is increased systemically in HF (Ito et al. 1994; Lehmann et al. 2014) and we previously showed that coronary vessels from EC‐MR^−/−^ have attenuated endothelin‐induced vasoconstriction (Mueller et al. 2015). Thus we determined the expression of endothelin‐1 and the endothelin receptors type A and B, in the LV of Sham and TAC EC‐MR^+/+^ and EC‐MR^−/−^ mice. Whereas the endothelin receptor B (*ednrb*) increased in the LV of EC‐MR^+/+^ mice in response to TAC, this increase was not observed in EC‐MR^−/−^ mice after TAC and remained similar to the expression observed in EC‐MR^−/−^ Sham mice (Fig. 6D). Conversely, endothelin receptor type A (*ednra*) expression did not change in response to TAC in EC‐MR^+/+^ mice but both this vasoconstrictive receptor, and expression of the ligand, endothelin‐1, were significantly decreased in EC‐MR^−/−^ TAC mice, as compared to EC‐MR^−/−^ Sham and EC‐MR^+/+^ TAC mice (Fig. 6E and F). Taken together, these data indicate that in the absence of EC‐MR, expression of endothelin‐1 and its receptors are decreased in the LV after TAC, supporting the potential for improved coronary function to contribute to preservation of systolic function.

## Discussion

In this study we investigated the role of MR in endothelial cells in the process of cardiac remodeling leading to HF using an EC‐specific MR‐KO mouse model with intact immune cell MR combined with the well‐established mouse model of HF induced by TAC. We observed that EC‐MR^−/−^ mice appear to be protected from the early stages of development of systolic dysfunction after TAC with relatively preserved ejection fraction and decreased expression of ANP and BNP in association with attenuated LV diastolic pressure overload compared to MR intact littermates. This protection occurred with no difference in cardiac hypertrophy LV MHC isoform switching, cardiac perivascular, and interstitial fibrosis, or indices of diastolic dysfunction. Also, the degree of cardiac angiogenesis and apoptosis were unchanged by deletion of EC‐MR. Contrary to our hypothesis, the absence of EC‐MR did not affect the increase in LV IL‐1*β* and IL‐6 cytokine expression or ICAM‐1 expression, and thus there was a similar degree of LV leukocyte infiltration as EC‐MR^+/+^ littermates in response to TAC. Rather, EC‐MR‐deficient mice had decreased LV expression of TNF*α*, endothelin‐1, and endothelin receptors A and B, compared with EC‐MR^+/+^ TAC mice. Overall, our study demonstrates for the first time that EC‐MR does not impact cardiac systolic or diastolic function during homeostasis but contributes to the progression from pathologic cardiac hypertrophy to systolic dysfunction in response to LV pressure overload induced by TAC.

MR antagonists reduce mortality in patients with HF with reduced ejection fraction (Pitt et al. 1999, 2003; Zannad et al. 2011). Despite the clinical benefit of MR antagonists, their specific cellular target(s) and the precise mechanism for these benefits have not yet been fully uncovered. A meta‐analysis of randomized clinical trials revealed that pharmacological blockade of MR has beneficial effects on cardiac remodeling and improves parameters of LV function in HFrEF patients (Li et al. 2013). Delineating the precise tissues through which MR modulates these effects has been an area of active investigation and overall suggests that MR signaling in different cell types plays distinct roles. While overexpression of MR in cardiomyocytes induces spontaneous development of classical features of HF such as cardiac hypertrophy and fibrosis (Le Menuet et al. 2001; Ouvrard‐Pascaud et al. 2005), cell‐specific deletion of MR has provided divergent results depending on the cell targeted and the HF model used. For example, MR deletion in cardiac myocytes did not affect cardiac hypertrophy or fibrosis in response to TAC but resulted in improved systolic function. This was in contrast to selective MR deletion in cardiac fibroblasts, which did not have any beneficial effect (Lother et al. 2011). Our new data indicate that deletion of EC‐MR does not affect systolic function during homeostasis, but contributes to the development of systolic dysfunction in response to TAC without modulating cardiac hypertrophy, fibrosis, or diastolic function. This outcome is similar to the findings in cardiac myocyte specific MR‐deficient mice, and supports that MR in EC and cardiac myocytes regulates aspects of pressure overload‐induced HF that are independent of these maladaptive cardiac remodeling features classically associated with the pathology of HF. These findings also raise the possibility that some of the benefits of MR antagonists in patients with cardiac dysfunction may arise independently of effects on cardiac fibrosis and hypertrophy.

A recent clinical trial of MR antagonism in HFpEF was negative (Pitt et al. 2014), indicating no benefit of MR inhibition in the global population enrolled in the trial. HFpEF is a heterogenous condition with multiple distinct etiologies, including metabolic heart disease from diabetes or obesity, pressure overload from hypertension or valve disease, aging‐associated cardiac stiffness, and infiltrative cardiomyopathies (Ferrari et al. 2015). Post hoc analyses support the hypothesis that MR inhibition may have benefit in subsets of patients with HFpEF (Pfeffer et al. 2015; Bristow et al. 2016). The specific populations that may benefit could depend on the etiology of HFpEF or the timing of treatment relative to the progression of disease. The role of EC‐MR in this process is only beginning to be explored. One study, using the same EC‐specific MR knock out mouse model, showed that cardiac stiffness and diastolic dysfunction induced by obesity was prevented in female EC‐MR^−/−^ mice (Jia et al. 2015). This study also suggests that while EC‐MR does not contribute to the degree of hypertrophy after pressure overload, it could play a role in the transition from compensatory hypertrophy to cardiac dilation and systolic failure. If so, the timing of treatment could be an important consideration in patients with HF. Further studies are needed in animal models, to clarify the mechanism by which EC‐MR contributes to HF and also in patients, to determine whether MR antagonists may be beneficial in subsets of patients with HFpEF for which there are no other efficacious therapies.

Our findings that EC‐MR deletion did not affect LV leukocyte infiltration after TAC were unexpected for several reasons. Prior work from our group and others demonstrated that EC‐MR regulates ICAM‐1 expression, which contributes to leukocyte adhesion to coronary endothelial cells (Caprio et al. 2008), a necessary step for cardiac leukocyte infiltration and that aldosterone‐induced vascular inflammation in atherosclerosis requires ICAM‐1 (Marzolla et al. 2017). Moreover, we and others have recently demonstrated that ICAM‐1 is necessary for T‐cell recruitment to the heart (Salvador et al. 2016) and that T cells are necessary for cardiac inflammation, hypertrophy, and fibrosis in HF induced by TAC, as T‐cell‐deficient mice are protected from these cardiac responses to pressure overload (Laroumanie et al. 2014; Nevers et al. 2015). We therefore predicted that EC‐MR would contribute to cardiac remodeling by promoting cardiac T‐cell infiltration, inflammation, and fibrosis. However, our findings demonstrate that EC‐MR in fact does not contribute to upregulation of IL‐1*β* and IL‐6 after TAC with no effect on ICAM‐1 upregulation and hence does not modulate leukocyte infiltration nor the associated cardiac fibrosis. These data additionally suggest the possibility that IL‐1*β* and/or IL‐6 may be responsible for inducing ICAM‐1 upregulation in the LV independently of MR signaling in HF (Luscinskas et al. 1991; Schnoor et al. 2015). These unexpected findings therefore provide novel insights suggesting that EC‐MR regulation of cell adhesion molecules to promote tissue inflammation may be context dependent occurring in the aorta in the presence of hyperlipidemia but not in the heart in response to TAC. Studies exploring the mechanism for this differential effect of EC‐MR on ICAM‐1 expression and inflammation are warranted.

Our findings are especially relevant in clarifying the interpretation of previous studies in which the Tie2 promotor drove MR deletion in both the EC and leukocyte lineages. For example, mice with MR deleted from both EC and leukocytes have decreased cardiac inflammatory infiltration and fibrosis in response to deoxycorticosterone/salt‐induced hypertension (Rickard et al. 2014). The lack of inflammatory attenuation in our selective EC‐MR deletion model, compared with the reduction in these processes in the previously published model could be due to a role for MR function in leukocytes, rather than in ECs, or to the distinct stimulus of DOCA/salt hypertension versus TAC. It is not clear whether the low expression of MR in T cells (Herrada et al. 2010) is sufficient to mediate such an effect, but one could speculate that this could be a potential mechanism. Additionally, we found that CD11b+ monocytes also infiltrate the LV of EC‐MR^−/−^ mice in similar numbers as in their wild‐type littermates exposed to TAC. Given the critical role of monocyte/macrophage MR in promoting macrophage polarization to the proinflammatory M1 phenotype and in the development of cardiac fibrosis in two separate models of hypertension‐induced cardiac remodeling (Rickard et al. 2009; Usher et al. 2010), our data support the notion that EC‐MR does not regulate cardiac fibrosis, but rather that MR in infiltrating leukocytes likely mediates this profibrotic effect. Indeed, we demonstrate that leukocyte infiltration in perivascular areas colocalize with perivascular fibrosis in EC‐MR^−/−^ and EC‐MR^+/+^ mice. While we found that ICAM‐1 is upregulated in response to TAC independent of EC‐MR in vivo*,* we report, for the first time to our knowledge, that the production of the proinflammatory cytokine TNF*α* is prevented in the LV of mice specifically lacking EC‐MR. This is in line with previous studies in which MR antagonism reduced plasma TNF*α* levels in rodent models of ischemic HF (Francis et al. 2003; Kang et al. 2006), however, a specific role for EC‐MR could not be determined from those studies. The lack of TNF*α* induction in the LV of EC‐MR^−/−^ in response to TAC could potentially explain the preserved systolic function as TNF*α* can directly contribute to cardiac dysfunction (Bozkurt et al. 1998).

Although EC‐MR^−/−^ and EC‐MR^+/+^ mice were exposed to the same degree of LV pressure overload and the development of similar cardiac hypertrophy and fibrosis, EC‐MR^−/−^ mice displayed an ejection fraction and fractional shortening very similar to Sham operated mice and preserved as compared to EC‐MR^+/+^ mice exposed to TAC. These changes in systolic function were modest at this point in the progression of TAC‐induced HF and other parameters of LV function and structure were similar in both genotypes. One possibility is that the improved phenotype of EC‐MR^−/−^ mice arises from an improved aortic compliance as a consequence of the lack of EC‐MR. However, this hypothesis is unlikely because effective arterial elastance did not differ between EC‐MR^+/+^ and EC‐MR^−/−^ littermates after TAC (Table 2). We considered the possibility of EC‐MR could contribute to cardiomyocyte death yet at 4 weeks TAC induces limited apoptosis (Li et al. 2009; Nevers et al. 2015), and TUNEL staining of LV sections was not different between TAC EC‐MR^+/+^ and TAC EC‐MR^−/−^ mice. Whether EC‐MR contributes to the degree of apoptosis at later times after TAC requires further exploration. Alternatively, EC‐MR may contribute to the cardiac functional response to pressure overload by modulating coronary blood flow. We recently reported that EC‐MR contributes to vascular contractile function specifically in the coronary vasculature at baseline and after exposure to hypertensive stimulus of angiotensin II infusion. Specifically, EC‐MR deletion attenuated coronary vasoconstriction in response to endothelin‐1 (Mueller et al. 2015), which is increased in HF (Ito et al. 1994; Lehmann et al. 2014). Thus, an intriguing possibility is that EC‐MR mediates endothelin‐1‐induced vasoconstriction of coronary vessels in TAC mice through its receptors ETA and ETB in the setting of pressure overload, thereby contributing to impaired coronary blood flow and resulting in systolic dysfunction. If so, the lack of EC‐MR may preserve coronary flow by preventing coronary vasoconstriction mediated by endothelin receptor signaling and therefore contributing to the maintenance of the ejection fraction. Our data indicating that the expression of endothelin‐1 and the endothelin receptor A are decreased in TAC EC‐MR^−/−^ mice versus TAC EC‐MR^+/+^ is in support of this mechanism since the endothelin A receptor is expressed on smooth muscle cells and mediates coronary vasoconstriction in response to endothelin‐1 (Luscher and Barton 2000). The endothelin B receptor is expressed on ECs and mediates vasodilation (Luscher and Barton 2000), and may be upregulated in compensation for enhanced vasoconstriction in HF. Studies have shown that MR in EC regulates endothelin receptor B expression (Mueller et al. 2015) and contributes to posttranslational modifications that attenuate endothelin B receptor function (Maron et al. 2012). Thus substantial additional studies are needed to determine the specific mechanism by which EC‐MR interactions with the endothelin signaling cascade and whether this contributes to cardiac dysfunction in response to pressure overload or other stimuli. Overall, the concept that MR contributes to cardiac dysfunction by regulating coronary vessel constriction or relaxation is in line with recent reports demonstrating that smooth muscle cell MR contributes to coronary dysfunction in mice subjected to experimental myocardial infarction (Gueret et al. 2016), that overexpression of MR in cardiomyocytes impairs coronary vasodilation (Favre et al. 2011), and with clinical data demonstrating that MR blockade improves coronary vascular function in patients with diabetes (Garg et al. 2015). However, since coronary function was not evaluated in these studies, this potential mechanism remains speculative and future studies are needed to test this novel hypothesis.

There are some other limitations to consider in this study. First, evidence from animal models and HF patients treated with MR antagonists support a contribution of the aldosterone‐MR axis in both systolic and diastolic dysfunction (Pitt et al. 1999, 2003; Zannad et al. 2011; Ayuzawa et al. 2016). Because EC‐MR^−/−^ are mainly protected from systolic dysfunction, MR expressed by other cell types could be the mediator of diastolic dysfunction, however, we have not evaluated this possibility in mice with multiple genetic deficiency of MR in other cells besides EC in the context of TAC. Additionally, for practical reasons, all experiments were performed only in male mice, and there is evidence that HF differentially impacts male and females (Blenck et al. 2016), and that estrogen can modulate the transcriptional activity of MR (Barrett Mueller et al. 2014). Thus additional studies are needed to characterize the role of EC‐MR in conjunction with MR in other cells and its role in female mice.

In conclusion, this study reveals that EC‐MR contributes to LV systolic dysfunction in response to pressure overload induced by TAC without affecting cardiac fibrosis or hypertrophy. Despite a known role for EC‐MR in promoting inflammation and a role for inflammation in progression of HF EC‐MR deletion also did not impact proinflammatory leukocyte recruitment in the TAC model of HF. These results, together with the growing body of literature evaluating MR signaling in other cell types involved in HF, enhance the global understanding of the role of MR in the development of HF. These findings further suggest that some of the beneficial effects of MR antagonists in improving cardiac function and preventing mortality in HF patients could be independent of effects on cardiac hypertrophy or fibrosis but rather could involve regulation of proinflammatory cytokines and/or endothelin signaling in the heart or coronary vasculature.

## Conflict of Interest

None.
